# Extended depth-of-field light-sheet microscopy improves imaging of large volumes at high numerical aperture

**DOI:** 10.1063/5.0101426

**Published:** 2022-10-20

**Authors:** Kevin Keomanee-Dizon, Matt Jones, Peter Luu, Scott E. Fraser, Thai V. Truong

**Affiliations:** 1 Translational Imaging Center, University of Southern California, Los Angeles, California 90089, USA; 2 Molecular and Computational Biology, University of Southern California, Los Angeles, California 90089, USA

## Abstract

Light-sheet microscopes must compromise among field of view, optical sectioning, resolution, and detection efficiency. High-numerical-aperture (NA) detection objective lenses provide higher resolution, but their narrow depth of field inefficiently captures the fluorescence signal generated throughout the thickness of the illumination light sheet when imaging large volumes. Here, we present ExD-SPIM (extended depth-of-field selective-plane illumination microscopy), an improved light-sheet microscopy strategy that solves this limitation by extending the depth of field (DOF) of high-NA detection objectives to match the thickness of the illumination light sheet. This extension of the DOF uses a phase mask to axially stretch the point-spread function of the objective lens while largely preserving lateral resolution. This matching of the detection DOF to the illumination-sheet thickness increases the total fluorescence collection, reduces the background, and improves the overall signal-to-noise ratio (SNR), as shown by numerical simulations, imaging of bead phantoms, and imaging living animals. In comparison to conventional light sheet imaging with low-NA detection that yields equivalent DOF, the results show that ExD-SPIM increases the SNR by more than threefold and dramatically reduces the rate of photobleaching. Compared to conventional high-NA detection, ExD-SPIM improves the signal sensitivity and volumetric coverage of whole-brain activity imaging, increasing the number of detected neurons by over a third.

Studies of biological systems, ranging from molecular and cellular to collections of cells and whole organisms, reveal the need to capture spatial and temporal dynamics of multiple components over time. Optical imaging is well matched to these demands in basic science, clinical research, and the diagnosis and treatment of human disease. Optimal imaging of biological dynamics requires that the relevant spatial, temporal, and energetic scales be captured in their natural settings as they take place. Selective-plane illumination microscopy (SPIM; light-sheet microscopy) is well suited to this challenge, as its unique strategy in selective illumination of only the focal plane enables high-contrast, fast, volumetric image acquisition with lower light exposure than more typical imaging tools such as confocal laser scanning microscopy.^1^ Since its re-introduction close to two decades ago,^2,3^ SPIM has seen many advancements and has been widely applied to problems ranging from chemistry to developmental biology and neuroscience.^1^

Despite the amazing capabilities of SPIM, its success has motivated further development to meet the challenges of evermore demanding biological specimens, especially those that are too fast, large, noisy, and light sensitive. The excitation of fluorescent labels in the specimen creates fundamental limits on temporal and spatial resolution. Even an ideal detector with infinite speed and zero noise must wait for enough photons to form an image with acceptable signal-to-noise ratio (SNR).^4,5^ Thus, fundamental to the performance of any microscope is balancing the collection of as many of the signal photons and achieving the best spatial resolution as possible. Given the isotropic nature of fluorescence emission, a high-numerical-aperture (NA) detection objective that captures the largest possible emission solid angle yields ideal signal-collection efficiency and resolution. However, the tight point-spread function (PSF) of a high-NA detection objective, while providing the desired lateral resolution, necessarily comes with a narrow axial depth of field (DOF) (∼1–2 *μ*m full-width at half-maximum; FWHM), which is often a poor match to the thickness of the excitation light sheet, particularly for imaging large samples.

The excitation light sheet dimensions are limited by the trade-off between its thickness and Rayleigh range (useful field of view; FOV) due to diffraction.^1,6–11^ To image dynamic systems having volumetric dimensions of hundreds of micrometers with cellular resolution,^12–15^ low-NA illumination is needed to produce light sheets that span a sufficiently large FOV. However, low-NA illumination produces thicker light sheets (∼4–5 *μ*m, or thicker), which excites fluorescence outside the narrow DOF of any high-NA detection objective. This mismatch results in not only loss of fluorescence photons but also inclusion of more out-of-focus background, degrading both light-collection efficiency and SNR.

To strike a better compromise between light sheet excitation and detection, we have developed ExD-SPIM (extended depth-of-field SPIM), an approach that stretches the PSF of the detection objective in *z*, creating a greater DOF with minimal loss in *x–y* resolution (Fig. 1). The detection objective DOF is extended to match the light-sheet thickness, exploiting the full benefits of plane illumination: maintaining optical sectioning, reducing background, and achieving greater photon utilization efficiency. The needed DOF extension is achieved through a simple modification of the detection path, adding a “layer-cake” phase mask^16,17^ conjugate to the pupil plane of the high-NA detection objective in a standard SPIM setup^18^ (supplementary material Fig. S1 and Methods Section B). The layer-cake phase mask divides the full pupil of the detection objective into multiple zones, where each zone is created from a layer in the mask. The fluorescence passing through each of the zones is incoherent with each other due to the path difference between successive layers being much larger than the coherence length of the fluorescence signal.^16,17^ Upon arriving at the camera, the fluorescence from individual zones forms independent PSFs, each of which has an axially equal and elongated profile. Importantly, because of the mutual incoherence, the individual PSFs incoherently superpose to yield an effective PSF with the DOF axially extended by a factor approximately equal to the number of layers in the phase mask^16,17^ (the supplementary material, Methods Section A and Fig. S2a). As the incoherent zones are independent of the fluorescence wavelength, the axially elongated PSF is wavelength-independent. Our method simply extends the DOF by several folds [Figs. 1(a) and 1(c)], sacrificing little lateral resolution [Fig. 1(c)] and requiring no post-processing—images can be readout and interpreted directly.

**FIG. 1.**
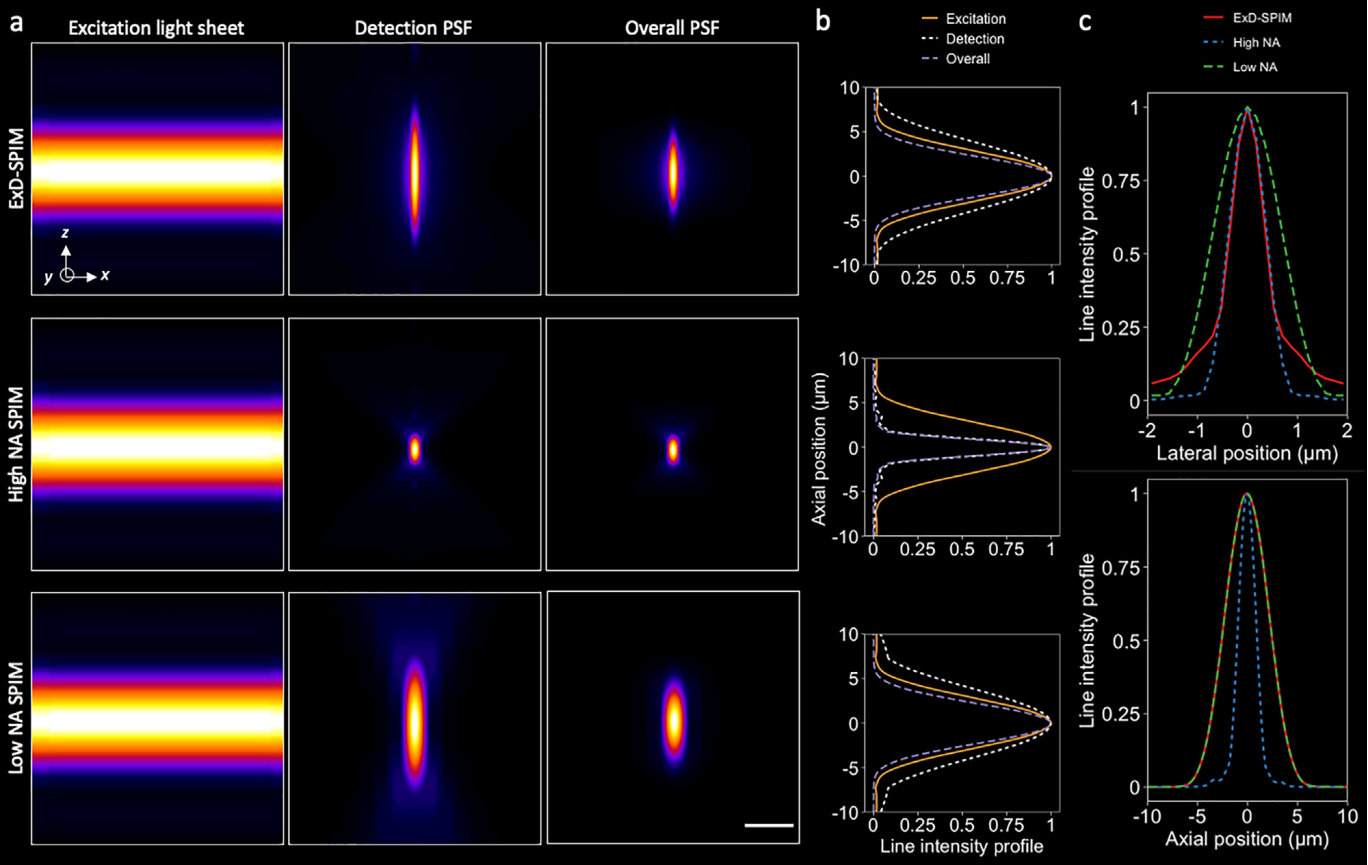
Instantaneous extended depth-of-field light-sheet microscopy at high numerical aperture. (a) Simulated 6-*μ*m-thick light sheets (left column) and detection PSFs (middle column) for ExD-SPIM (top row; NA=0.8), conventional high-NA SPIM (middle row; NA=0.8), and low-NA SPIM (bottom row; NA=0.41). Overall PSFs (right column) are calculated by multiplying the excitation PSF with the detection PSF. Scale bar, 5 *μ*m. (b) *x–z* cross-sectional intensity profiles of the excitation, detection, and overall PSFs. High-NA SPIM (middle row) provides maximal axial confinement of the overall PSF. As a result, however, only a fraction of the excitation light sheet results in the useful signal. ExD-SPIM (top row) captures the entire light-sheet thickness by instantaneously extending the DOF of the high-NA detection objective lens, just like a low-NA system (bottom row), but without massively degrading the lateral resolution (c). (c) Lateral (top) and axial (bottom) line intensity profiles of the overall PSFs of the modalities shown in (a).

**FIG. 2.**
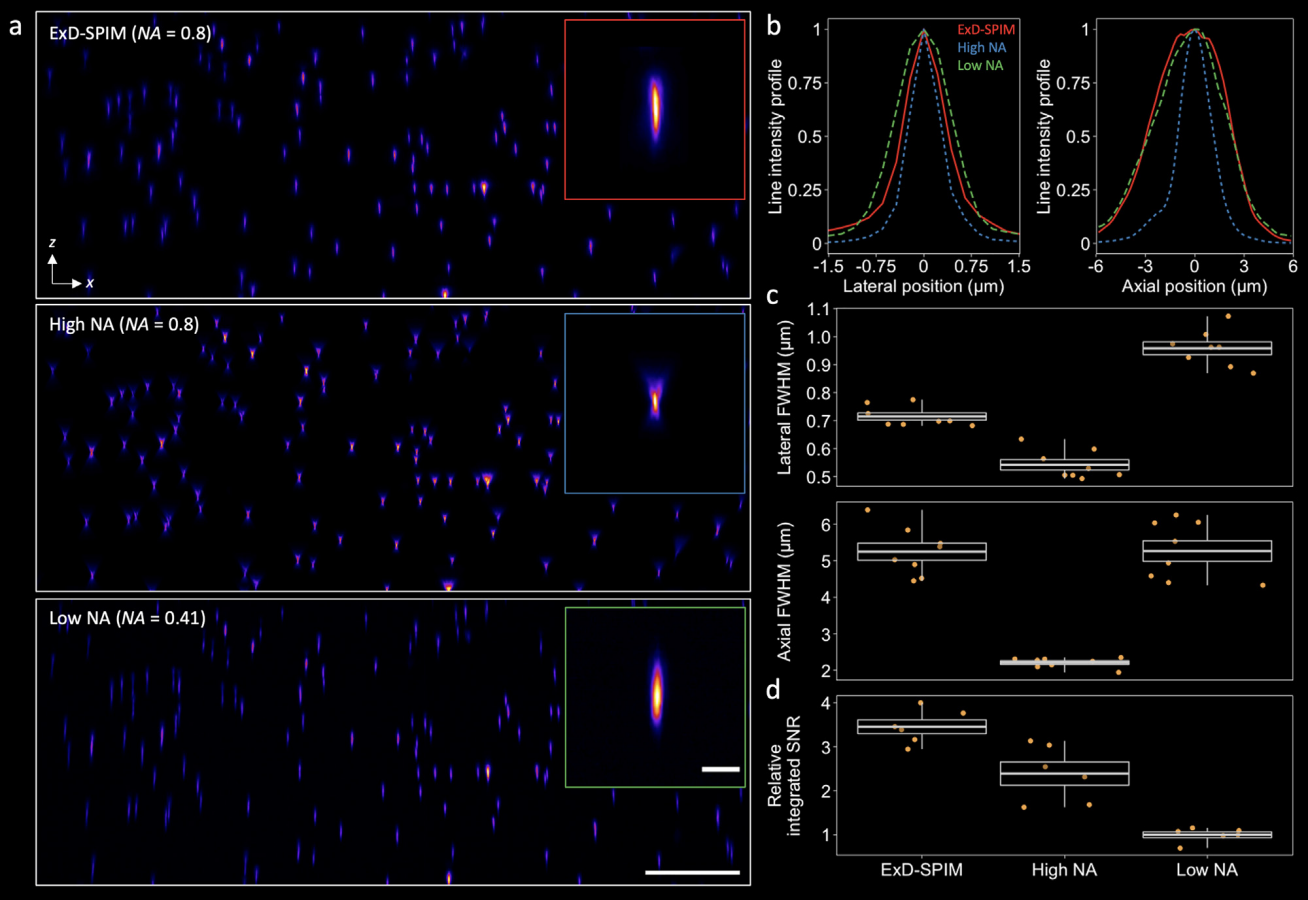
Experimental demonstration of improved light-sheet collection with instantaneous depth-of-field extension. (a) *x–z* maximum intensity projection of a 375 × 555 × 150 (*x–y–z*) *μ*m^3^ beads field captured with ExD-SPIM (top row), high-NA SPIM (middle row), and low-NA SPIM (bottom row). Inset: Representative PSFs computed by averaging over four beads for each modality. Gamma was adjusted to 0.75 for all modes. Scale bar, 50 *μ*m and (inset) 5 *μ*m. (b) Lateral (left) and axial (right) line intensity profiles through the PSFs in the insets of (a). ExD-SPIM shows an axial extent equivalent to the low-NA system with slight lateral blurring. (c) Lateral (top) and axial (bottom) FWHM measurements; for each modality, the same (*N *=* *8) eight beads were chosen from (a). The mean lateral and axial FWHM ± SD values are ExD-SPIM, 0.71 ± 0.04 and 5.25 ± 0.66 *μ*m, respectively; high NA, 0.54 ± 0.05 and 2.21 ± 0.14 *μ*m, respectively; low NA, 0.96 ± 0.06 and 5.26 ± 0.8 *μ*m, respectively. (d) Quantification of the integrated SNR (along both the *x* and *z* directions; see the supplementary material, Methods Section D) across multiple beads from an *x–z* summed-intensity projection of (a). ExD-SPIM shows ∼3.5× enhancement compared to low-NA SPIM and ∼45% enhancement over high-NA SPIM in the integrated SNR. All boxes are standard deviations; center values are means; whiskers represent the spread of the data.

ExD-SPIM is simpler, faster, and more robust than other SPIM techniques that elongate the DOF by hundreds of micrometers.^18–22^ DOF extension through wavefront coding^19–21^ requires computational transformation and deconvolution and loses in signal sensitivity at higher spatial frequencies. This makes dim features even dimmer and, thus, nonoptimal in low SNR regimes. Extending the DOF by introducing spherical aberration through the detection objective^22^ is only practical at low NA, which limits spatial resolution and light collection. Both of the above methods are affected by wavelength and can, thus, complicate multicolor fluorescence imaging. Increasing the DOF with an active optical device (electrically tunable lens,^23^ mirror galvanometer,^24^ or deformable mirror^25^) by remotely scanning the focus over an extended axial range during a camera exposure yields a time-averaged effective extended DOF but at low duty cycle. (Given fluorescence lifetimes are in the nanosecond range, and the fastest devices are currently slower by over an order of magnitude.) These axial scanning approaches capture the signal with far less efficiency and are limited in temporal resolution, compared to the instantaneous capture of photons over the extended axial range of ExD-SPIM.

Numerical simulations of ExD-SPIM validate the performance of our approach. We compared ExD-SPIM (NA=0.8) to conventional SPIM at high NA (NA=0.8) and SPIM at low NA (NA=0.41). The conventional high-NA mode serves as the high-resolution, diffraction-limited reference, while the low-NA mode serves as the low-resolution, DOF-equivalent reference. The PSFs were computed using the Debye approximation, as described by Chen *et al.*,^26^ with the overall PSF of each case calculated as the product of the detection PSF and a Gaussian-beam light-sheet with 6-*μ*m FWHM thickness (see the supplementary material, Methods Section A). We chose this light sheet thickness to experimentally target the imaging of neuronal nuclei (6–8 *μ*m in size) throughout the ∼400 × 800 × 250 (*x–y–z*) *μ*m^3^ brain of zebrafish larva at five days post-fertilization (dpf)^15^ (as will be shown below). We chose a four-layer-cake phase mask to extend the DOF by fourfold (from the intrinsic high-NA DOF of 2 *μ*m) in order to capture the entire light-sheet thickness across the field of view, so that all illuminated fluorophores are instantly in focus and their emitted photons imaged with high contrast [Figs. 1(b) and 1(d), the supplementary material, Methods Section A and Fig. S2(a)].

We experimentally benchmarked ExD-SPIM performance by measuring the PSF with sub-diffractive fluorescent beads embedded in a mixture of 1.5% agarose and 10% iodixanol.^27^ Compared with the high-NA reference (0.8 NA; N40X-NIR, Nikon, water-immersion objective lens), the ExD-SPIM PSF was accompanied by slightly broader shoulders in the lateral plane but lost only ∼30% in average lateral resolution across the imaged beads volume, whereas the low-NA system achieved significantly worse lateral resolution [Figs. 2(a) and 2(b)]. ExD-SPIM extends the PSF by ∼2.4-fold axially, compared to the high-NA system, comparable to the low-NA case [Figs. 2(a) and 2(b)]. The measurements of axial and lateral resolution are in good agreement with theoretical simulations with minor spatial asymmetry in all the PSFs, particularly for the high-NA and ExD-SPIM cases, likely due to the sensitivity to imperfections in the optical train and sample-induced aberrations [Figs. 2(b) and 2(c)]. Importantly, we can see a significant increase in the integrated SNR (the supplementary material, Methods Section D) of beads measured over a ∼375 × 555 × 150 *μ*m^3^ volume: ExD-SPIM SNR = 3.45 ± 0.38 (mean ± SD), conventional high-NA SPIM SNR = 2.39 ± 0.65 (ExD-SPIM ∼45% better), and low-NA SPIM SNR = 1 ± 0.16 [ExD-SPIM ∼350% better; Fig. 2(d)].

The larger DOF of ExD-SPIM compared to the smaller DOF of conventional high-NA SPIM leads to a direct benefit in high-speed volumetric imaging, allowing a sparser axial sampling interval in ExD-SPIM, provided that the extended DOF (and hence the axial resolution) is adequate for the target application. As demonstrated by recording a volume of fluorescent beads (Fig. S3), fewer *z*-slices are required by ExD-SPIM to reveal the same underlying features within the 3D volume (so long as the sampling satisfies the Nyquist limit, which requires that the *z*-spacing be no larger than half of the axial resolution). This improves the ability of ExD-SPIM to capture specimens with faster dynamics over larger volumes and reduces the total laser illumination, resulting in less photodamage.

**FIG. 3.**
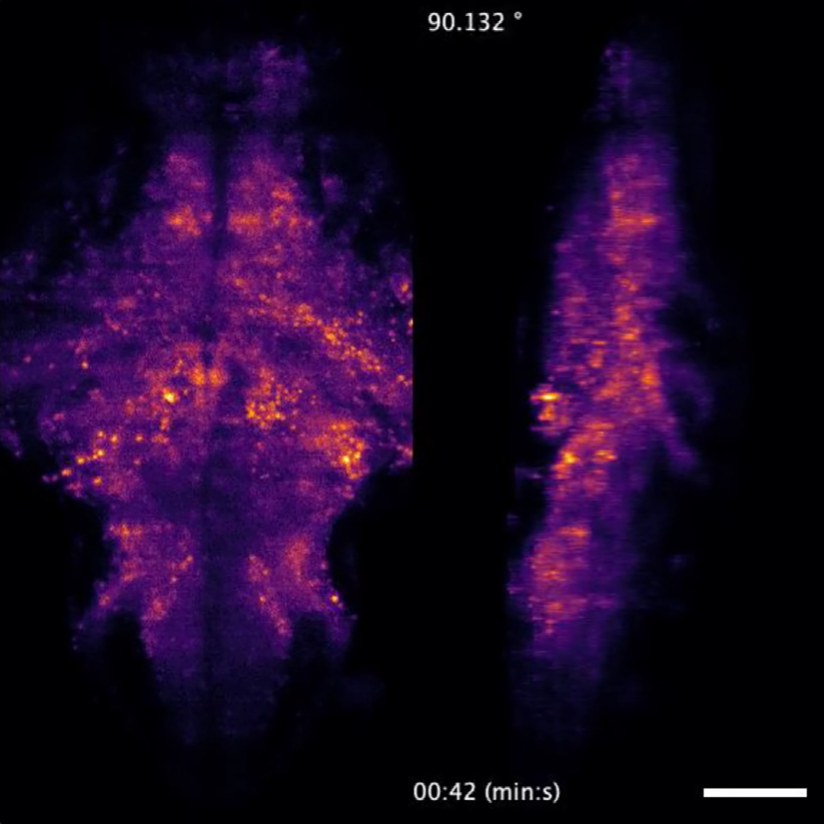
ExD-SPIM recording of the whole-brain neural activity. Dorsoventral (left) and rotating (right) maximum-intensity projections of a time-series recording of the whole-brain of a 5-dpf transgenic larval zebrafish. Whole-brain functional light-sheet imaging was performed at a volumetric rate of 5.64 Hz. The video is part of the data presented in Fig. 4. Scale bar, 100 *μ*m. Multimedia view: https://doi.org/10.1063/5.0101426.1
10.1063/5.0101426.1

The higher SNR of ExD-SPIM for each 2D optical section permits imaging using lower laser excitation, resulting in lower photodamage. We experimentally validated this by comparing rates of photobleaching during the recording of a single-plane of the fluorescent signals from GFP-labeled vasculature in live larval zebrafish (4–5 dpf). For comparison, the laser power was adjusted to achieve similar SNR in the first image of the time series; exposure times and other imaging parameters were identical over the different samples (the supplementary material, Methods Section C). ExD-SPIM performed the best: after 4 h of continuous imaging (70 760 images), the cumulative bleaching loss was only ∼5% of the initial fluorescence (Fig. S4). The bleaching loss of low-NA SPIM was ∼20%, ∼fourfold faster (Fig. S4). This demonstrates that ExD-SPIM achieves the twin goals of minimizing excitation light exposure and maximizing fluorescence collection for long-term *in vivo* imaging.

**FIG. 4.**
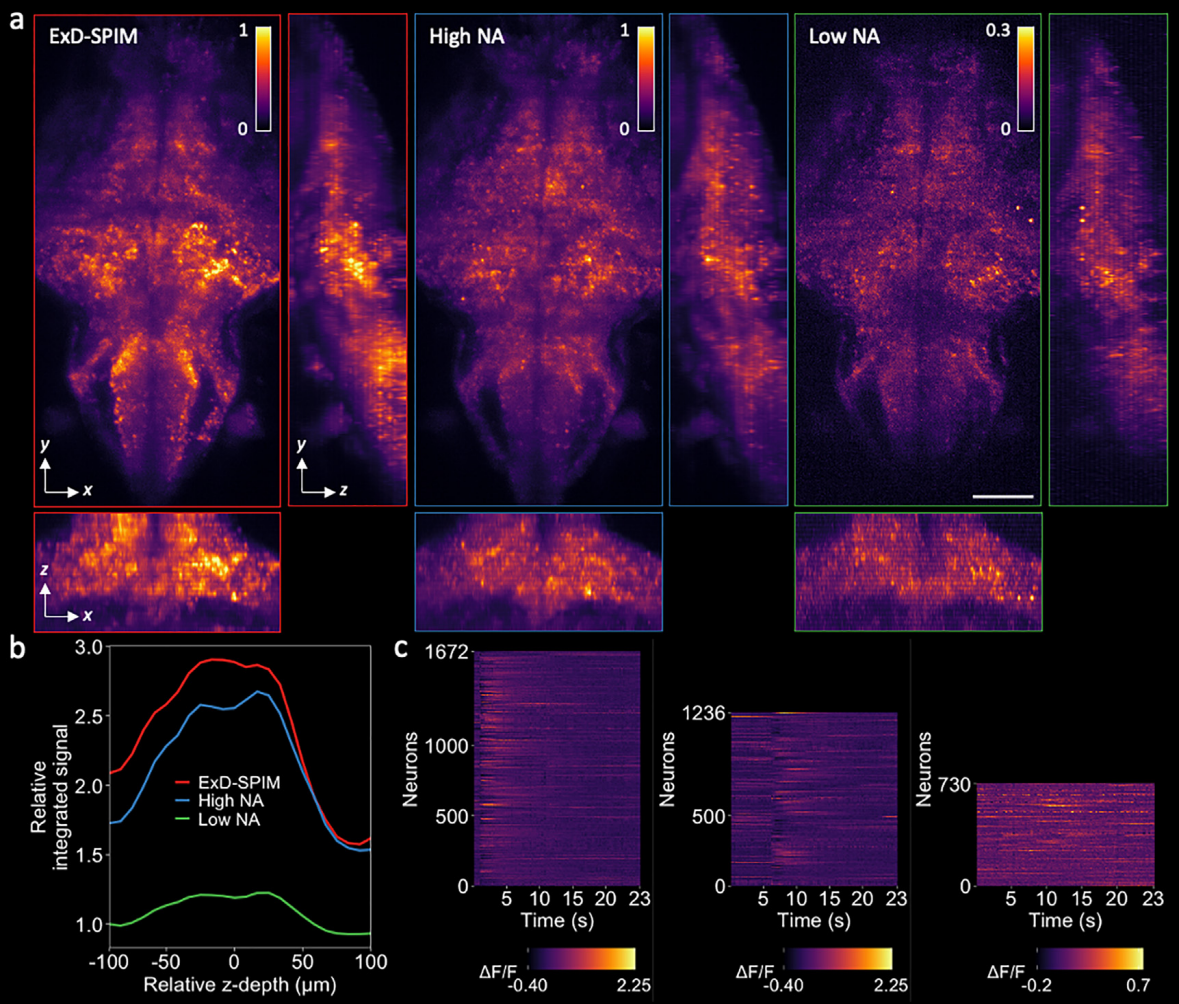
ExD-SPIM improves whole-brain activity imaging. (a) *x–y*, *y–z* (right), and *x–z* (bottom) maximum intensity projections (MIPs) of the spontaneous neural activity in transgenic larval zebrafish [*Tg*(*elavl3:H2b-GCaMP6s*)] at 5-dpf, captured by (left to right) ExD-SPIM (red), high-NA SPIM (blue), and low-NA SPIM (green). Imaging performed at 5.64 Hz of the same animal (the supplementary material, Methods Section C). Projections show the increased signal collection of ExD-SPIM compared to high-NA and low-NA SPIM. Scale bar, 100 *μ*m. Figure 3 shows a 3D rendered movie. (b) Plot shows the total summed intensity of the standard deviation projection [the same volume as in (a)] over a 5-min time series (1692 time points) as a function of the *z*-depth. The total integrated signal intensity is improved across the entire image volume with ExD-SPIM compared to SPIM at high NA and low NA. (c) Single-neuron activity traces captured by the different modalities, extracted over a 23-s window from the 4D time-series data of (b), indicating more neurons detected with ExD-SPIM than conventional SPIM at both high and low NA. ΔF/F computed as $(F-F_{0})/F_{0}$, where F is the mean fluorescence intensity and $F_{0}$ is the bottom eighth percentile of fluorescence over time.

To test ExD-SPIM on a dynamic volumetric imaging challenge, we imaged the spontaneous whole-brain activity in a 5-dpf transgenic larval zebrafish expressing the calcium indicator GCaMP6s. ExD-SPIM allowed us to collect volumetric images of 25 optical sections with high SNR, spanning 200-*μ*m in depth, at more than 5 Hz [Figs. 3 and 4(a)]. This volumetric imaging rate of ∼10^5^ voxels/s was limited only by the readout speed of the detector, not the available signal (Fig. 3, Multimedia view). To quantify the integrated signal and determine the number of active cells successfully captured in the time series, we used data collected over 5 min (1692 time points) of the same sample from each modality; this large sampling distribution (1695 images per *z*-plane) should average out any potential variance in the spontaneous brain activity between the different modalities. A 3D volumetric map of the temporal standard deviation for each *x–y* image plane of the 4D data highlights the positions of the active neurons and allowed us to extract single-neuron temporal traces.^18,28^ ExD-SPIM provided an overall increase in the total integrated signal at every acquired *x–y* image slice, by ∼11% compared to high-NA SPIM, and ∼220% compared to low-NA SPIM [averaged over the 25 optical sections; Fig. 4(b)]. More importantly, ExD-SPIM detected ∼35% more active neurons in the volumetric time series, collecting several hundreds of intracellular calcium transients that otherwise would have gone undetected with conventional high-NA detection [Fig. 4(c)]. These results highlight the improvements offered by ExD-SPIM for cellular resolution recording in challenging preparations.

Taken together, the above analyses demonstrate that ExD-SPIM optimizes the use of the photon budget, collecting more fluorescence and detecting more features for the same amount of excitation. The layer-cake phase mask used here introduces a slight drop in the peak intensity, but this loss is more than offset by the increased integrated signal collected axially. The use of the phase mask permits us to take advantage of the improved signal collection with increasing NA (roughly proportional to NA^2^) but without the more narrow DOF that typically accompanies increasing NA.^29^ Compared to low-NA detection, increasing the NA does increase the system’s sensitivity to optical aberrations and to instrument misalignment. Further, the highly inclined light rays associated with high-NA detection, that are uncollected by a low-NA objective, must travel longer distances from the plane of excitation through to the collection objective, increasing the susceptibility to both aberrations and scattering, especially deep in large, optically heterogenous specimens. Operation at higher NAs will require care in alignment and might benefit from adaptive optics to compensate for aberrations and achieve near-diffraction-limited performance.^30^

In this work, we have considered the imaging performance of different detection DOFs for a given light sheet thickness (and the corresponding FOV that it provides). This is motivated by the typical SPIM imaging experimental design that begins by considering the required FOV, which in turn defines the light-sheet thickness. If, instead, axial resolution is paramount then a different strategy would be employed. Given that the overall PSF is equal to the product of the detection PSF and the light-sheet profile (i.e., illumination PSF)^1,32^ [demonstrated in Fig. 1(a)], there are two natural regimes to consider. In the thin light-sheet regime, where the sheet thickness is thinner than the detection DOF, the overall axial resolution is mainly driven by the light-sheet thickness—this is the subcellular imaging regime where specially engineered light sheets are deployed.^8,11^ In this regime, changing the detection PSF by extending its DOF will yield minimal effect on the overall axial resolution. However, there could be practical benefits to having an extended DOF that is larger than the illumination-sheet thickness: it will be easier to align the light sheet to overlap the detection DOF and to maintain this alignment during 3D imaging of samples with inhomogeneous indices of refraction that could cause the light sheet to deviate from its original position. In the thick light-sheet regime, the sheet thickness is larger than the detection DOF, and the axial resolution is more dependent upon the detection DOF. This is the regime often involving large samples that our work is focused on, and where we have demonstrated the benefits of matching the detection DOF to the light-sheet thickness.

Our work shows that extending the detection DOF of a high-NA objective lens to match the light-sheet thickness allows us to reap the usual benefits of high NA while also getting enhanced signal collection and contrast. Of course, there are settings in which the lower axial resolution that comes from this extended DOF is not acceptable for the imaging application. In future developments of ExD-SPIM, it may be useful to “tune” the DOF extension, so that the user can select the desired balance between axial resolution vs signal, optical sectioning, and FOV from different light-sheet thicknesses. This could be achieved by implementing ExD-SPIM with multiple layer-cake phase masks, each with a different DOF extension (via different number of layers), mounted in a rotating turret, akin to a filter wheel changer, permitting switching between different masks to achieve the desired imaging parameters. We envision future SPIM setups should have the capability of adjusting its detection DOF (typically not available presently) in addition to the capability of adjusting its light sheet thickness (often provided in existing implementations). This will enable imaging across scales, spanning a wide range in space and time on the same light-sheet instrument, similar to the ability of confocal microscopy to adjust the detection pinhole size to the dimensions of the excitation focus.

The implementation of ExD-SPIM explored here is one of many ways to achieve an extended and optimized detection DOF. As an alternative to the transmission phase mask used in our work, ExD-SPIM could be implemented by an all-reflective pupil-plane mirror that induces the appropriate path length differences between multiple zones to generate an incoherent superposition of the foci at the image plane (the supplementary material, Methods Section A). Alternatively, a deformable mirror might be employed to simultaneously perform DOF extension, axial *z*-scanning, and adaptive optics correction. This would require a deformable mirror with sufficiently large size, stroke, and speed to accommodate the proper phase modulations.

We have demonstrated that ExD-SPIM offers a more optimal compromise for light-sheet imaging of large specimens, providing an important alternative to the reduced performance of moderate-to-low NA optics of most instruments designed for imaging of large samples.^9–11,31^ The improved photon efficiency offered by extended DOF at high NA could be used to boost the imaging speed and temporal resolution without needing to increase the laser excitation power (and thus avoiding potential increased photodamage). Alternatively, ExD-SPIM could permit imaging with significantly less laser excitation power, generating the same SNR with less photodamage, which is critical for studying light-sensitive systems or cellular dynamics over long timescales.^33,34^ The simple modification needed to accomplish ExD-SPIM using a pupil phase mask avoids the expense, extensive optical redesign, or the wavelength-dependent phase modulation of many approaches, making it easily adaptable to multicolor fluorescence microscopy and scalable for high-throughput imaging.

See the supplementary material for detailed methods (theory and numerical simulations, microscope optics, sample preparation and imaging conditions, and quantifying signal-to-noise ratio); the schematic of the extended depth-of-field light-sheet microscope (Fig. S1); the theoretical principle and simulations of extended-detection point-spread functions (Fig. S2); ExD-SPIM enabling reduced axial sampling rate in volumetric imaging (Fig. S3); and ExD-SPIM offering low photodamage *in vivo* (Fig. S4).

## Data Availability

The data that support the findings of this study are available from the corresponding author upon reasonable request.
